# Omega-3 Fatty Acid Deficiency in Infants before Birth Identified Using a Randomized Trial of Maternal DHA Supplementation in Pregnancy

**DOI:** 10.1371/journal.pone.0083764

**Published:** 2014-01-10

**Authors:** Kelly A. Mulder, D. Janette King, Sheila M. Innis

**Affiliations:** Nutrition and Metabolism Program, Child and Family Research Institute, Department of Paediatrics, Faculty of Medicine, University of British Columbia, Vancouver, British Columbia, Canada; Iran University of Medical Sciences, Islamic Republic of Iran

## Abstract

**Background:**

DHA is accumulated in the central nervous system (CNS) before birth and is involved in early developmental processes, such as neurite outgrowth and gene expression.

**Objective:**

To determine whether fetal DHA insufficiency occurs and constrains CNS development in term gestation infants.

**Design:**

A risk reduction model using a randomized prospective study of term gestation single birth healthy infants born to women (*n* = 270) given a placebo or 400 mg/day DHA from 16 wk gestation to delivery. Fetal DHA deficiency sufficient to constrain CNS development was assessed based on increased risk that infants in the placebo group would not achieve neurodevelopment scores in the top quartile of all infants in the study.

**Results:**

Infants in the placebo group were at increased risk of lower language development assessed as words understood (OR 3.22, CL 1.49–6.94, *P* = 0.002) and produced (OR 2.61, CL 1.22–5.58, *P* = 0.01) at 14 mo, and words understood (OR 2.77, CL 1.23–6.28, *P = *0.03) and sentences produced (OR 2.60, CL 1.15–5.89, *P* = 0.02) at 18 mo using the McArthur Communicative Developmental Inventory; receptive (OR 2.23, CL 1.08–4.60, *P = *0.02) and expressive language (OR 1.89, CL 0.94–3.83, *P = *0.05) at 18 mo using the Bayley Scales of Infant Development III; and visual acuity (OR 2.69, CL 1.10–6.54, *P = *0.03) at 2 mo.

**Trial Registration:**

ClinicalTrials.gov NCT00620672

## Introduction

Nutrient deficiencies during development may have long-lasting consequences for the central nervous system (CNS) that range from devastating malformations to subtle effects on neural functioning. Persisting CNS deficits due to early deficiency depend on the nutrient and inadequacy at a time vulnerable to morphological or molecular shifts from which recovery is difficult [1], [2]. In this regard, docosahexaenoic acid (DHA, 22:6n-3), but not other n-3 fatty acids, is enriched in brain and retina membranes where it functions in early developmental events such as neurogenesis, neurite outgrowth, synaptic plasticity, axonal elimination, and gene expression [3]–[11]. Loss of CNS DHA is compensated for by increased 22:4n-6, 22:5n-6 and sometimes arachidonic acid (ARA, 20:4n-6) in animals and human infants [5], [12]–[14]. Although substitution of n-6 fatty acids for DHA fulfils needs for membrane fatty acids, n-6 fatty acids differ in functional properties from DHA. This results in a complex problem whereby the consequences of insufficient DHA may reflect both loss of DHA and the actions of the n-6 fatty acids used in replacement.

Understanding dietary needs for n-3 fatty acids for CNS development is complicated by the different n-3 fatty acids in the diet, interactions with n-6 fatty acids, and genetic and other variables that impact their metabolism. The n-3 fatty acids are consumed as α-linoleic acid (18:3n-3), mainly in plant oils, and eicosapentaenoic acid (20:5n-3) and DHA from animal lipids, particularly fish. Conversion of 18:3n-3 to DHA occurs in humans, but in a complex pathway requiring Δ-6 desaturase, which has at least 6 substrates [15], [16]. Among these, n-6 fatty acids are important due to the abundant 18:2n-6 in westernized diets, inhibition of 18:3n-3 desaturation by high 18:2n-6, and knowledge that 20:4n-6, 22:4n-6 and 22:5n-6 accumulate, and hence n-6 fatty acid desaturation occurs, in infants and animals fed diets high 18:2n-6 [13], [14], [17], [18]. When consumed, DHA effectively provides DHA to the developing CNS thus overcoming concerns over 18:3n-3 inadequacy, ability to synthesize DHA, or high n-6 fatty acid intakes [13], [19]–[22].

Our work focuses on whether n-3 fatty acid deficiency, leading to DHA low enough to compromise fetal CNS development occurs in gestation. Observation studies to show a positive association between child CNS development and maternal DHA intake or blood DHA levels in pregnancy [23]–[26] provide evidence that insufficient DHA to support child CNS development does occur in some pregnant women. Randomized clinical trials on the efficacy of supplemental DHA in pregnancy in enhancing child CNS performance have yielded mixed findings [27]–[37], but whether the infants in these studies were compromised by underlying n-3 fatty acid insufficiency before birth is unknown.

The present study is based on a risk-reduction model, and used a randomized intervention with DHA or a placebo to address whether fetal DHA supplies are sufficiently low in some pregnant women as to increase risk that children will not meet their developmental potential. The design recognizes that neither the prevalence nor extent of DHA insufficiency, if present, is known. The efficacy of DHA in enhancing cognitive development can only be tested in individuals known to have deficits in cognitive development. For example, if it is accepted that infants fed formula have cognitive delays compared to breast-fed infants, then the efficacy of DHA in preventing the cognitive deficits due to feeding formula can be tested by comparing infants fed formula with and without DHA. Equally important, our design incorporates understanding that the placebo group in this study is not a non-exposure group. Variable amounts of DHA are present in the diet of all subjects. Further, individuals in the DHA supplement group who are not deficient are not expected to benefit. Because our underlying goals are to demonstrate deficiency and subsequently identify biomarkers of deficiency, maternal blood samples were a pre-requisite to assessment of child outcomes in the present work.

## Subjects and Methods

### Ethics Statement

This study was conducted according to the guidelines laid down in the Declaration of Helsinki and all procedures were reviewed and approved by the Committee for Ethical Review of Research Involving Human Subjects at the University of British Columbia (B.C.) and the B.C. Children's and Women's Hospital. Written informed consent was obtained from each woman prior to randomization on behalf of herself and for her infant. The protocol for this trial and supporting CONSORT checklist are available as supporting information; see Checklist S1 and Protocol S1.

### Study Design, Intervention and Subjects

**Figure 1:**
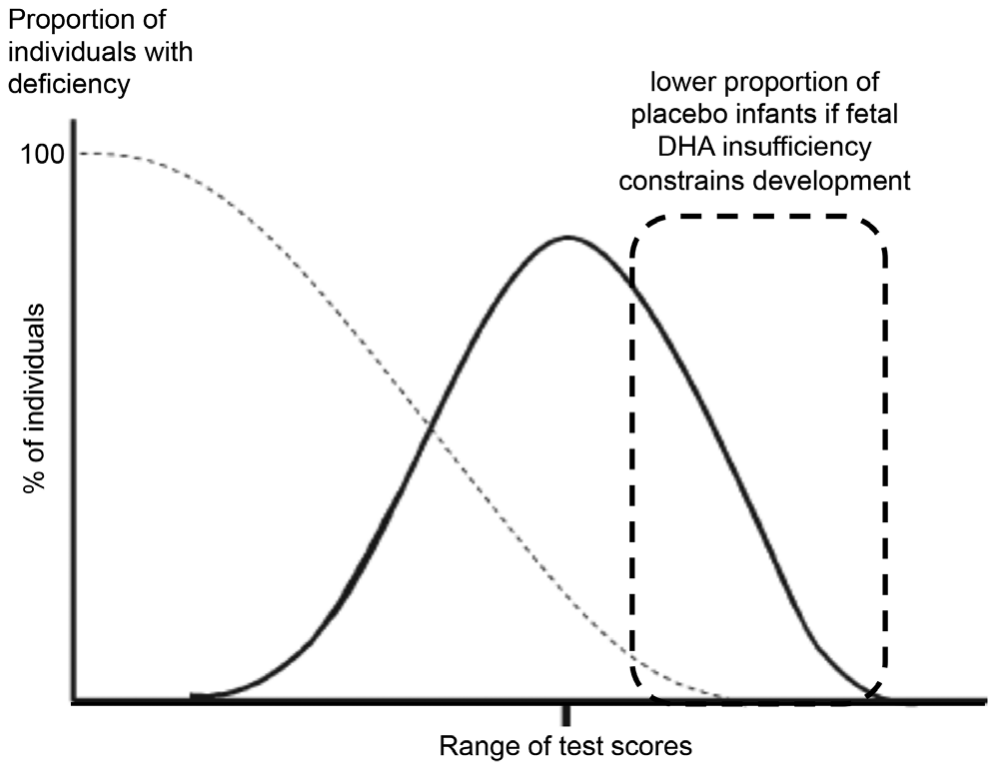
Schematic to illustrate the concept that individuals with high neurodevelopment test scores are unlikely to be nutrient deficient. We assessed whether fetal DHA insufficiency sufficient to constrain CNS development occurs based on failure to achieve a neurodevelopmental test score in the upper 25% of infant scores, representing the range of achievement where deficiency is less likely.

This was a double-blind, single center, randomized study designed to assess whether DHA was sufficiently low among some term infants prior to birth as to limit CNS development to 18 mo-of-age. All of the subjects were residents of Vancouver, Canada and enrolled between 2004 and 2008. The purpose of randomizing a group of women to DHA was to provide a group of infants in which risk of DHA insufficiency during gestation was considered low. The rate of CNS development differs among infants, giving a distribution of scores within which the best potential development of an individual infant is unknown. As a result, the risk reduction model focused analyses on performance of infants in the placebo group within the distribution of development of all infants in the study. We hypothesize that prenatal DHA insufficiency will constrain child CNS development. If this occurs, we expect that fewer infants in the placebo than DHA supplement group will achieve high neurodevelopmental test scores, giving evidence that DHA deficiency occurs during gestation and constrains child development in our population (**Figure 1**). As described [38], [39], our studies were not designed to test the efficacy of DHA supplements in increasing child CNS abilities, which is a different question and not our hypothesis. Efficacy of an intervention can only be addressed with individuals known to be able to respond to the intervention; in our situation the presence of deficiency and thus the ability to respond is unknown. In the event that no effect is found, the interpretation is that DHA deficiency was not detectable in our study population, not that DHA is not needed by the brain. An assumption of the prevalence of DHA deficiency was used for the purpose of study design. We assumed DHA deficiency in 15% of infants before birth and 35% dropout, with increased risk of not being in the 25% of infants with the highest test scores identified in the placebo group with enrolment of 270 subjects, *P*<0.05.

Eligible participants were ≤16 wk gestation, not taking any lipid or fatty acid supplement, and were expected to deliver one infant at full-term gestation, with no maternal or fetal complications. After enrolment, subjects were assigned to 400 mg/d DHA or a placebo using computer-generated, random codes held in sealed, opaque envelopes using a block design. The supplements were provided in identical capsules in bottles with more than sufficient supplements to cover the study interval. Compliance was monitored from the number of returned unused capsules. DHA was given as algal oil triglycerides and the placebo was an equivalent amount of corn and soybean oil blended to reflect the dietary 18:2n-6 and 18:3n-3 ratio, but in amounts quantitatively insignificant compared to usual intakes [38]. The supplements were identical in appearance, contained an orange flavour mask and were provided by DSM Nutritional Products (formerly Martek Biosciences, MD). We chose a supplement of 400 mg/d DHA since this is equivalent to DHA from about two meals of fatty fish/wk, and four to ten-fold higher than the amount of DHA accumulated/d in fetal tissues [40], [41]. Higher amounts of DHA would give greater separation between the intake and blood levels of DHA [42] among women in the DHA and placebo supplement groups. However, this is not a useful strategy for this study because of the underlying understanding that providing more DHA than required by the CNS will not result in enhanced CNS function. Fundamentally, the relationship between the intake of an essential nutrient and functions dependent on that nutrient are non-linear, with a linear dose-response between DHA and child cognitive function unlikely. We do not hypothesize that DHA supplements increase cognitive abilities in individuals who fulfill their DHA requirements. The duration of supplementation was from enrolment until infant delivery. Maternal venous blood and information on dietary intake was obtained at 16 wk gestation prior to commencing supplements, then again at 36 wk gestation.

**Figure 2:**
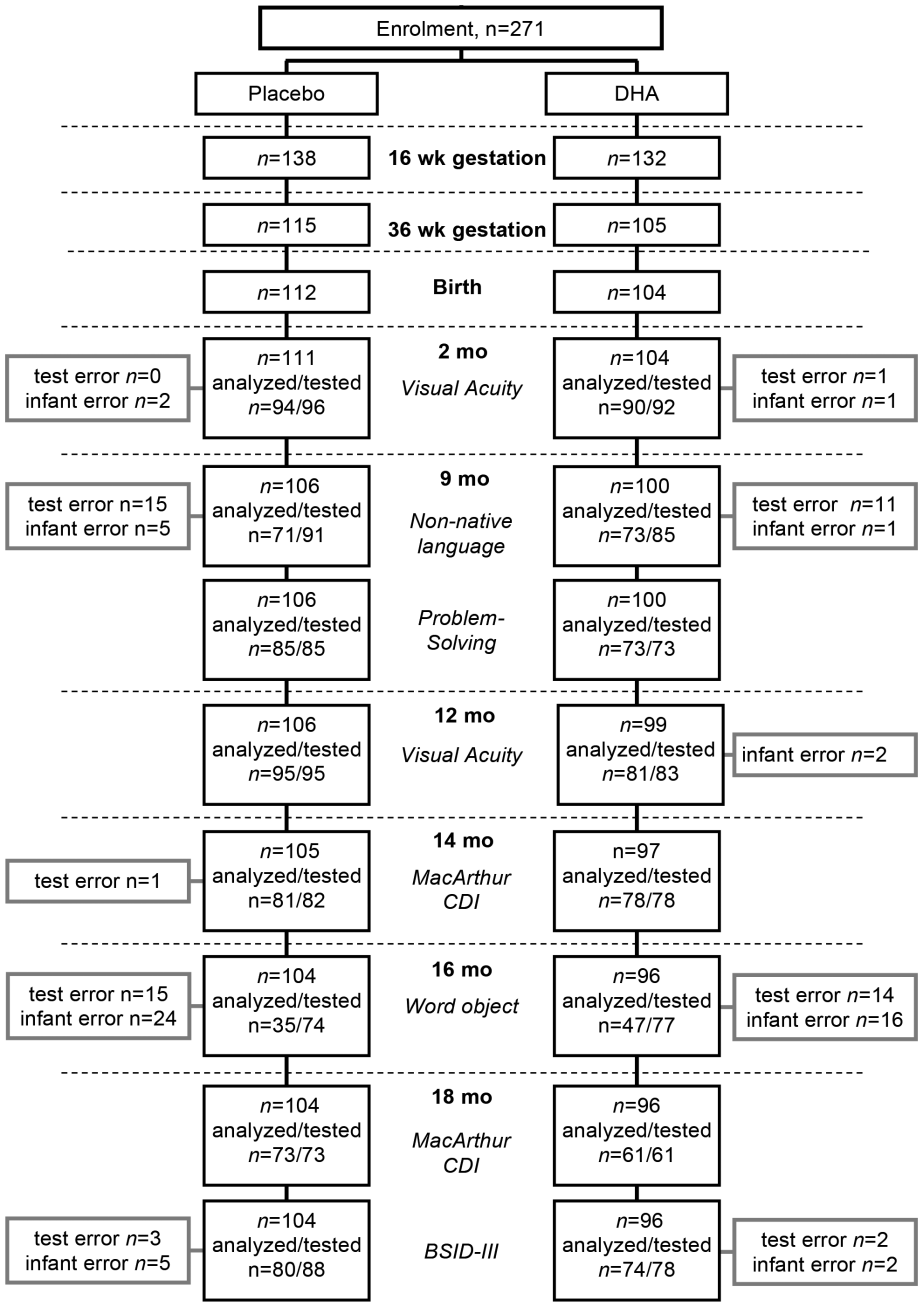
Flow diagram of study participants and their infants from enrolment to completion. Subject withdrawal from 16 to 36*n* = 9, 16; protocol non-compliance, *n* = 7, 6; preterm delivery, miscarriage, elective pregnancy termination or other pregnancy complications, *n* = 5, 5; and one woman in the placebo group delivered twins, lost 36 wk blood sample *n = *1, 0. One subject in the placebo group withdrew between 36 wk gestation and infant delivery, with self-withdrawal and loss to follow-up of infants between 2 and 9 mo of age of *n* = 5,4; between 9 and 12 mo of age of *n* = 0,1; between 12 and 14 mo of age *n* = 1,2; between 14 and 16 mo of age *n* = 1,1; and between 16 and 18 mo of age *n* = 0, 0 for the pacebo and DHA groups, respectively. Three infants born at term gestation did not meet the protocol requirements for follow up, explained by congenital disorders, *n* = 1,1 and intrauterine growth retardation, *n* = 1,0 in the two groups, respectively, and one infant in the placebo group died from sudden infant death syndrome before 2 mo of age. After birth, the number of infants at each milestone, with the number of infants attending assessments, and number of tests incomplete or not analyzed is given. For the placebo and DHA groups, respectively, infant tests done but not analyzed were: at 2 mo, *n* = 0,1 technical error, *n* = 2,1 uncooperative infant; at 9 mo non-native contrast test, *n* = 15,11 technical error, *n* = 4,0 uncooperative infant, *n* = 1,0 did not habituate, *n* = 0,1 exposed to Hindi at home; at 12 mo visual acuity, *n* = 0,2 uncooperative infant; at 14 mo, *n* = 1,0 unresolved parent reporting error; at 16 mo, *n* = 15,14 technical error, *n* = 18,16 uncooperative infant, *n* = 6,0 did not habituate; and at 18 mo, BSID-III, *n* = 3,2 technical error and *n* = 5,2 uncooperative infant. Technical errors included video-recording and test protocol errors, or external disruption, such as noise causing infant distraction; infant errors include fussy or uncooperative behaviour, and parent interference.

A flow diagram of subject enrolment, retention in the study, infant developmental tests and reasons tests were not analyzed are in **Figure 2**. Two hundred and seventy one pregnant women completed the informed consent. One woman withdrew before commencing the study, leaving 138 and 132 women in the placebo and DHA groups, respectively. Blood was obtained and analyzed for 114 and 103 women in the DHA and placebo groups, respectively, at both 16 and 36 wk gestation. Infants of mothers who did not complete the 36 wk prenatal assessment or had complications, including prematurity, likely to interfere with normal development were not included in the follow-up. Maternal socio-demographic characteristics, including age, ethnicity, family income, education, and parity were recorded. Each mother's IQ was assessed using the Test of Nonverbal Intelligence-3 (TONI-3), which assesses aptitude, abstract reasoning and problem solving [43]. Data on infant birth weight, pregnancy weight gain, and gestation length were collected from hospital charts. Infant weight and length was measured at 2, 6, 9, 12 and 18 mo of age.

### Dietary and Biochemical Analyses

Venous blood samples were centrifuged, the plasma and buffy coat recovered, and the red blood cells (RBC) washed in saline and centrifuged three times. All samples were stored frozen at -80°C until analyses. Phosphatidylethanolamine (PE) is predominately located on RBC inner membrane. As in the brain, the RBC PE contains higher DHA, 20:4n-6, 22:4n-6 and 22:5n-6 than phosphatidylcholine (PC), and shows considerably less variability in response to recent change in fatty acid intake than plasma lipids or RBC PC [44]–[46]. Therefore, RBC PE and PC were separated by HPLC, and their fatty acids analyzed by GLC [13], [21], [22], [47], [48]. Maternal dietary intake was assessed at 16 and 36 wk gestation using a food frequency questionnaire, then analyzed for macronutrient and fatty acid intakes [47]. Infant feeding, including breast-feeding and breast-feeding duration, was collected monthly at a study visit or by telephone if no visit occurred. For this study, breast-feeding was defined as exclusive as long as the intake of formula did not exceed 250 mL/wk.

### Assessments of Infant Development

Visual acuity was assessed using the Teller Acuity card procedure at 2 mo ±3 d and 12 mo ±3 d using test distances of 38 cm and 55 cm, respectively [48]. Language development was assessed using four approaches: a non-native consonant recognition task at 9 mo ±3 d [48]; the MacArthur Communicative Development Inventory (CDI) infant scale at 14 mo ±5 d and infant and toddler scales at 18 mo ±5 d; a word-object pairing task at 16 mo ±5 d [49], and Bayley Scales of Infant and Toddler Development, Third Edition (BSID- III) language composite scales at 18 mo ±5 d. Problem solving ability was also assessed at 9 mo ±3 d of age [50], and the BSID-III cognitive, fine and gross motor scales were given at 18 mo ±5 d. The non-native consonant recognition and problem solving tests were videotaped, then the tapes coded and later scored by observers unaware of the infant's study group. The acceptable age range during which each developmental assessment was made was purposefully narrow, based on the premise that DHA insufficiency constrains the rate of child CNS development.

### Data Analyses

All data were analyzed on a per protocol basis. Subject characteristics were summarized using descriptive statistics, then compared using independent two-sample t-tests for continuous variables, Mann-Whitney *U* tests for nonparametric continuous variables, and chi-square for categorical data. Data was checked for skewness using the Shapiro-Wilk test of normality prior to analysis. Z-scores for infant weight-for-age, length-for-age and weight-for-length were calculated using the World Health Organization (WHO) Anthroplus anthropometric calculator software (version 1.0.4). Maternal RBC fatty acids and dietary intakes at 16 and 36 wk gestation were analyzed using two factor ANOVA and Wilcoxen signed-rank test, respectively. Spearman's Rho were used to assess the association between maternal dietary intake of DHA and the RBC PE and PC DHA. Logistic regression was used to assess maternal and infant variables impacting infant test results, and these included maternal age, maternal IQ, maternal RBC DHA at enrolment, infant birth weight, gestation length, infant sex, birth order, and breast-feeding duration. Smoking was not included because only 6 women reported that they smoked at any time during pregnancy. Of the variables assessed, only infant sex differed between the two randomized groups and showed a potential relationship to infant outcome (*P*≤0.05); therefore, sex was included in all subsequent analyses.

The odds ratio and 5-95^th^ confidence interval (CI) that an infant in the placebo or DHA group would fail to perform among the top 25% of all infants was determined with contingency tables and Fisher exact test. For some tests, separation of the cohort into an exact upper 25% and lower 75% of infants was not possible when more than one infant achieved the same test score at the group cut point. In those cases, we defined the upper 25% of infants as the n that gave the lowest possible deviation from n = 25% of all infants tested for that test. Pass/fail results for the problem-solving and non-native language consonant tests were compared between the DHA and placebo supplement groups using chi square. Data analysis was conducted with IBM SPSS Statistics (Version 20.0.0, 2011. Chicago, IL) and differences considered significant at a *P* value <0.05.

## Results

Of the 138 and 132 women randomized to the placebo and DHA groups, 22 in the placebo group and 27 in the DHA group withdrew before 36 wk gestation (**Figure 2**). Subject withdrawal was explained for the two groups respectively by subject self withdrawal, *n* = 9 and 16; protocol non-compliance, *n* = 7 and 6; preterm delivery, miscarriage, elective pregnancy termination or other pregnancy complications, *n* = 5 and 5; and one woman in the placebo group delivered twins. One blood sample was lost for one woman in the placebo group at 36 wk gestation due to technical error, giving 115 and 105 women in the placebo and DHA groups, respectively, for whom RBC fatty acids were analyzed at 36 wk gestation (**Figure 2**). Diet records were analyzed for all subjects with the exception of one woman in the DHA group at each time point. Two blood samples collected at 16 wk gestation were not analyzed due to technical error. One subject in the placebo group withdrew between 36 wk gestation and infant delivery. Three infants born at term gestation did not meet the protocol requirements for follow up, explained by congenital disorders, *n* = 2, and intrauterine growth retardation, *n* = 1, and one infant in the placebo group died from sudden infant death syndrome before 2 mo of age. Retention in the study after birth exceeded 90%, with 111 and 104 infants in the placebo and DHA groups, respectively, at 2 mo of age and 104 and 96 infants in the two groups, respectively, at 18 mo of age. The number of infants in the study at 2, 9, 12, 14 and 18 mo of age, with the number of infants attending assessment visits, tests analyzed, and reasons tests were not analyzed are in Figure 2.

The characteristics of the 115 and 105 women in the placebo and DHA group, respectively, who completed the study protocol to 36 wk gestation did not differ for maternal age, IQ, or pregnancy weight gain (**Table 1**). The women were primarily of Caucasian background (73.6%), most with post-secondary education (94.4%). No differences in maternal and family socio-demographic characteristic were found between the randomized groups. For the 216 infants at delivery (Figure 2), 113 were girls and 103 were boys. For reference, on average 105 boys are born for every 100 girls in Canada. Since DHA cannot alter sex, it was by chance that there were more infant boys (*P*<0.02) in the placebo than DHA group. Infant weight, length and weight-for-length z-scores did not differ between the two randomized groups at any age (see **Table S1 in File S1**). Breast-feeding rates were high, with 77% and 70% of the placebo group, and 72% and 63% of the DHA group infants fed <250 mL infant formula at 4 and 6 mo-of-age, respectively, (*P*<0.05).

**Table 1:** Maternal and infant characteristics classified by intervention group^1^.

	Maternal group	
	Placebo *n* = 111	DHA *n* = 104
Maternal age (y)	33.4±3.61	32.6±4.04
Ethnicity, white/non-white (%)	73.9/26.1	73.1/26.9
Parity, % 1, 2, >2	47.7, 36.7, 15.6	60.6, 30.8, 8.6
Pre-pregnancy weight (kg)^2^	64.7±12.6	64.8±12.6
Pregnancy weight gain (kg)	14.7±4.48	14.1±4.82
Maternal IQ^3^	34.0±7.27	34.4±7.58
Infant sex, % boys/girls^4^	55.0/45.0	40.4/59.6
Infant birth weight, (g)	3497±479	3494±400
Infant birth weight (z score)^5^	0.36±1.02	0.42±0.78

^1^ Values are mean ± SD or % as shown for infants eligible for follow-up and their mothers.

^2^ Pre-pregnancy weight was by self-report.

^3^ Maternal IQ was assessed using the Test of Non-Verbal Intelligence-3 (TONI-3) (37).

^4^ There were more boys in the placebo than the DHA group, *P* = 0.03, with a trend to less first-born infants in the placebo than DHA group, *P* = 0.06, by Chi-square.

^5^ Birth weight was not available from hospital charts for 2 infants in the DHA group (*n = *102).

The maternal dietary n-6 and n-3 fatty acid intakes did not differ between the groups at 16 or 36 wk gestation (see **Table S2 in File S1**). Briefly, fat provided 34.2±6.60 and 33.5±5.74% dietary energy at 16 wk gestation and 34.7±6.46 and 34.7±5.55% dietary energy at 36 wk gestation, for women in the placebo and DHA groups, respectively (*P*>0.05). DHA intakes were skewed, with a median (2.5 to 97.5^th^ percentile range) intake in the placebo (*n* = 115) and DHA (*n* = 104) group, respectively, of 80.0 (0.00-334) and 90.0 (6.00-472) mg/d at 16 wk and 90.0 (10.0-302) and 100 (10.0-346) mg/d at 36 wk gestation (*P*>0.05). Fish (including shellfish) intakes were 141 (0-546) and 182 (0-708) g/wk at 16 wk gestation and 170 (0-570) and 168 (0-885) g/wk at 36 wk gestation for the placebo and DHA groups, respectively (*P*>0.05).

**Figure 3:**
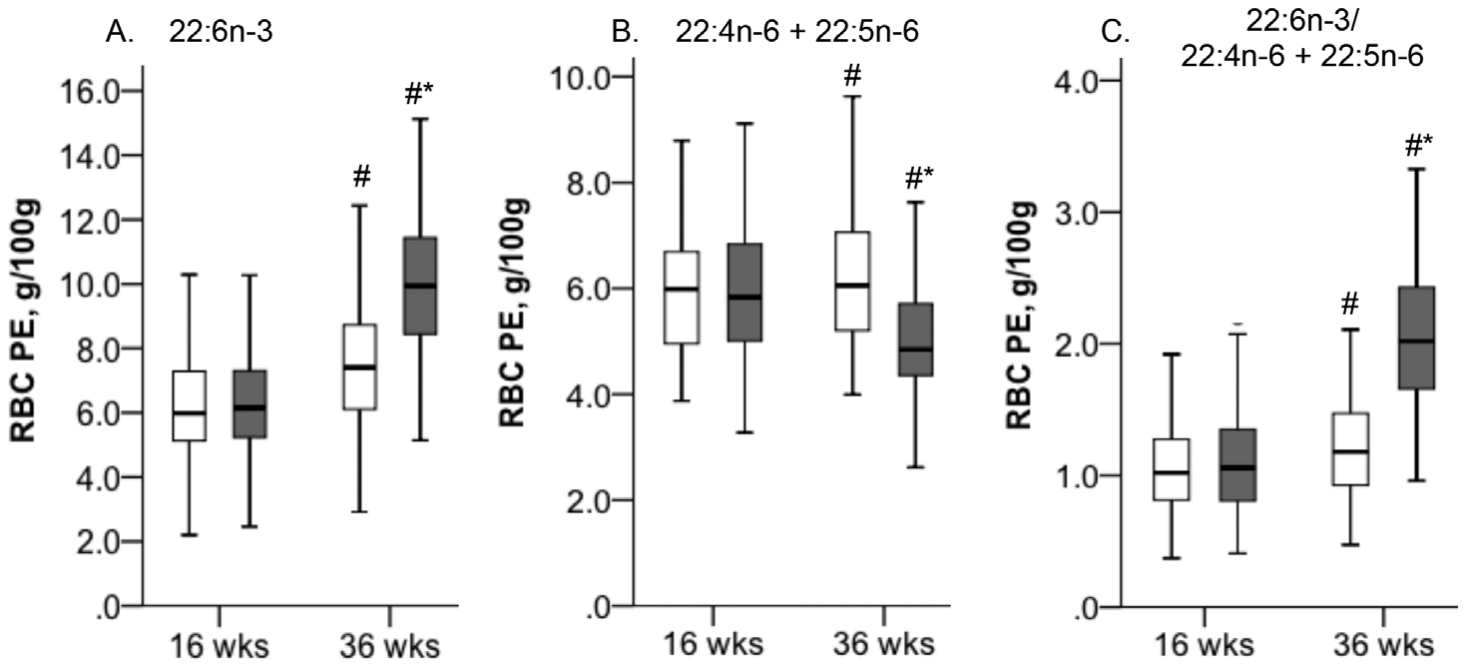
Boxplots to show maternal red blood cell (RBC) phosphatidylethanolamine (PE) g/100 g fatty acids for women assigned to a placebo (open bars) or supplement of 400 mg/d DHA (closed bars) from 16 wk gestation until infant delivery. Panels A, B and C are the RBC PE DHA, 22:4n-6+ 22:5n-6, and ratio of DHA/22:4n-6+ 22:5n-6, respectively; *n* = 111 and 111for the placebo group, and *n* = 102 and 104 for the DHA group at 16 and 36 wk gestation. # Value at 36 wk gestation different from 16 wk gestation within a group, *value for placebo group different from DHA group at the same stage of gestation, *P*<0.01, by two way ANOVA.

The maternal RBC PE DHA was 6.25±1.60 and 6.36±1.62 g/100 g fatty acids in the placebo and DHA groups, respectively, at 16 wk gestation (*P*>0.05), with a significant increase to 10.0±2.06 g/100 g at 36 wk gestation in the DHA group (*n* = 105, *P*<0.01) compared to 7.40±2.04 g/100 g fatty acids in the placebo group (*n* = 115, *P*<0.01) (**Figure 3**). In contrast, the RBC PE 22:5n-6 increased from 16 to 36 wk gestation in the placebo group (*P*<0.01), while 22:5n-6 and 22:4n-6 decreased from 16 to 36 wk gestation in the DHA group (*P*<0.01). Regardless of the difference in the mean levels of DHA in the RBC PE between the groups at 36 wk gestation (*P*<0.01), the range of RBC PE DHA showed overlap between the groups. Specifically, the 2.5-97.5^th^ percentile range of RBC PE DHA was 3.52 to 11.2 and 6.09 to 14.0 g/100 g fatty acids for women in the placebo and DHA groups, respectively, at 36 wk gestation (Figure 3). Levels of ARA were not different between the groups at 16 wk gestation, but ARA decreased in the RBC PE in DHA group to levels below the placebo group at 36 wk gestation (*P*<0.05, see **Table S3 in File S1**). Results for the analyses of the maternal RBC PC fatty acids are provided in **Table S4 in File S1**. Dietary DHA intake was positively associated with DHA levels in the maternal RBC PE and PC among all women at 16 wk gestation (rho 0.341, rho 0.346, *P*<0.001, respectively, *n* = 214) and in the placebo group at 36 wk gestation (rho 0.286, rho 0.330, *P*<0.001, respectively, *n* = 111) (see **Figure S1**).

Analyses for categorical results for visual acuity at 2 and 12 mo-of-age, the pass/fail results for the problem-solving, non-native language consonant contrast test at 9 mo-of-age, and the word-object learning task at 16 mo-of-age are described first, followed by the results for the continuous variables (scores) for the CDI and BSID-III. Less than 50% of infants completed the word-object learning task at 16 mo-of-age, likely because of the test duration, and results for this test were not further analyzed. Using the results, we defined high visual acuity as an acuity ≥3.3 cycles/degree at 2 mo-of-age and ≥13 cycles/degree at 12 mo-of-age (**Table 2**). Infant girls showed a non-significant trend to higher visual acuity than boys at 2 mo (*P* = 0.07), but not 12 mo-of-age (*P* = 0.79). Infants in the placebo group were at increased risk of not achieving a visual acuity ≥3.3 cycles/degree at 2 mo of age (OR, 2.50, CI 1.02–6.14, *n* = 184, *P* = 0.03), with no evidence of increased risk of failure to achieve high visual acuity at 12 mo of age (OR, 1.23, CI 0.61–2.49, *n* = 176, *P* = 0.35, Table 2). Analysis of the pass/fail results for the problem-solving at 9 mo of age found more girls (65%) than boys (49%) were successful (*P = *0.03). However, there was no difference in success between the placebo and DHA groups when stratified by sex for infant girls (*P* = 0.21) or boys (*P* = 0.27). Similarly, we found no difference in ability to discriminate the non-native language consonant between boys and girls, or between the placebo and DHA groups, with 44.9% (*n* = 78) of girls and 43.9% (*n* = 66) of boys (*P* = 0.91), and 45.1% of infants in the placebo and 43.8% in the DHA group (*P* = 0.88) able to discriminate the non-native consonant contrast at 9 mo of age.

**Table 2:** Risk that an infant in the placebo group would fail to be among infants achieving high visual acuity^1^.

Agemo	Acuity threshold cycles/degree	%Placebo/DHA above acuity threshold	Odds ratio (CI)	*P*
2	≥3.3	8.51/18.9	2.50 (1.02–6.14)	0.03
12	≥13	21.1/24.7	1.23 (0.61–2.49)	0.35

^1^ High visual acuity was defined as a visual acuity ≥3.3 and ≥13 cycles/degree at 2 mo and 12 mo of age, respectively. Odds ratios and 5^th^-95^th^ confidence interval (CI) determined using contingency tables with Fisher exact analysis.

**Figure 4:**
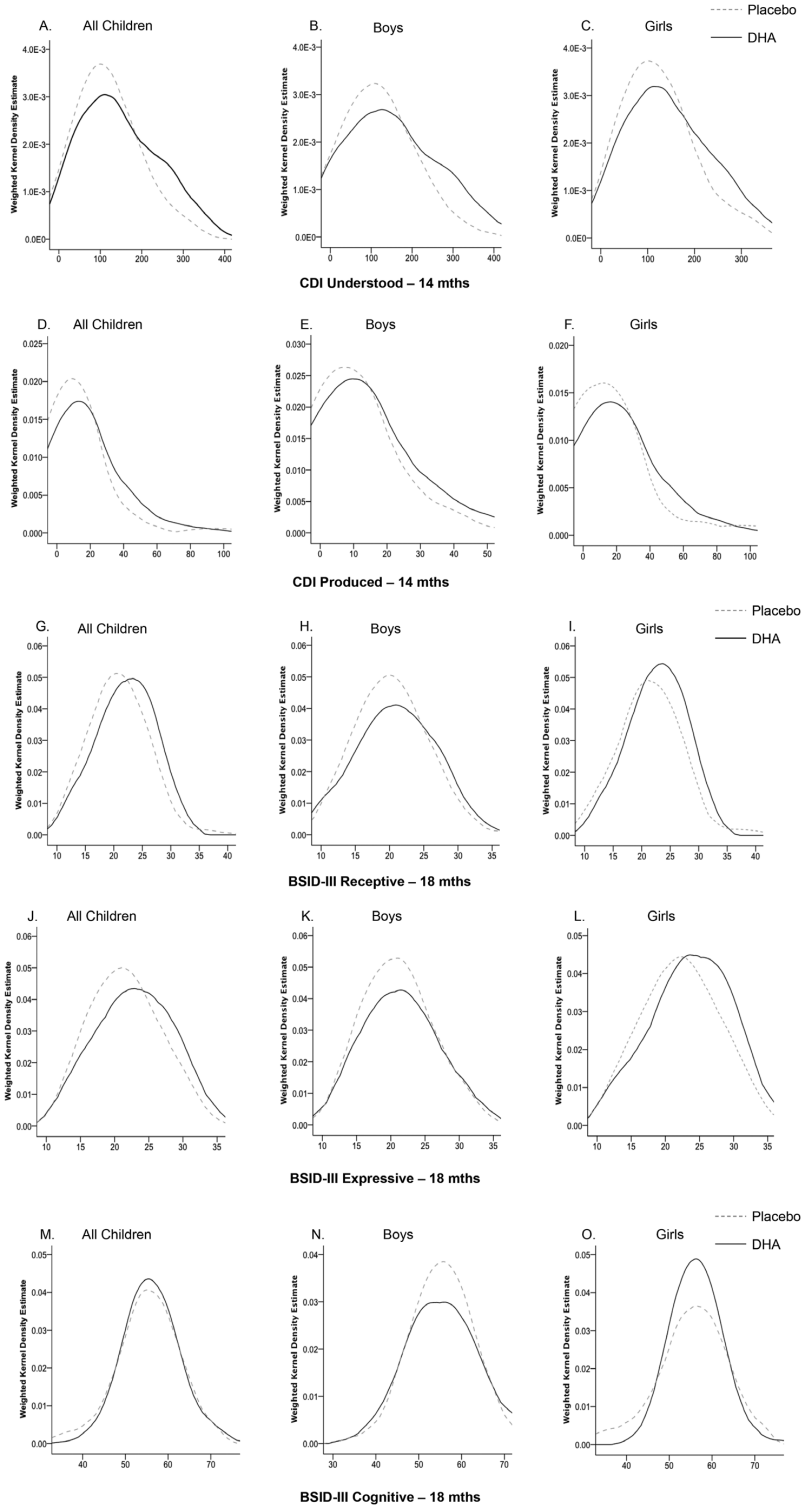
Representative weighted Kernal density plots to show examples of the distributions of test scores for the DHA and placebo groups for all infants, boys and girls for outcomes in which maternal DHA decreased risk of not achieving high development: CDI, communicative developmental inventories and BSID, Bayley Scales of Infant development receptive and expressive language in panels A-L, or had no effect, BSID-III cognitive in panels M-O.

Infant sex was significantly related to the performance on the CDI and BSID-III. Girls scored higher than boys on the CDI infant scale for words produced at both 14 and 18 mo of age (*P* = 0.02, *P* = 0.01, respectively), on the CDI infant scale for words understood at 14 mo of age (*P* = 0.02), and the CDI toddler scale of words produced (*P* = 0.01) and the BSID-III receptive (*P* = 0.03) and expressive (*P* = 0.02) language scales at 18 mo of age. There was no significant difference between boys and girls on the BSID-III cognitive, fine motor or gross motor scales (*P*>0.05). Representative frequency plots illustrating the distributions of test scores in all infants, boys and girls in the DHA and placebo groups on languages tests at 14 and 18 mo of age, and the BSID-III cognitive subscales for which effects of DHA were or were not found, respectively, are in **Figure 4**. Infants in the placebo group were at increased risk of not performing in the highest 25% of infants for words understood (OR 3.22, CI 1.49–6.94, *P* = 0.002) and produced (OR 2.61, CI 1.22–5.58, *P* = 0.01) at 14 mo of age, and for words understood (OR 2.77, CI 1.23–6.28, *P = *0.03) and sentences produced (OR 2.60, CI 1.15–5.89, *P* = 0.02), with a similar trend for words produced (*P* = 0.07) at 18 mo of age (**Table 3**). Infants in the placebo group were also at increased risk of not performing in the highest 25% of infants on the BSID-III receptive language (OR 2.23, CI 1.08–4.60, *P = *0.03) and expressive language scales (OR 1.89, CI 0.94–3.83, *P = *0.05) at 18 mo of age. We found no evidence of increased risk that girls or boys in the placebo group would not be among the 25% of infants with the highest scores on the BSID-III cognitive (*P = *0.70), fine motor (*P = *0.33) or gross motor skill subscales (*P = *0.40) at 18 mo.

**Table 3:** Risk that an infant in the placebo group would fail to achieve high scores on tests of language, cognitive and motor skill development^1^.

Test	Placebo/DHA in highest quartile	Age	OR (CI)	
	%	*mo*		*P*
**Infant CDI** 2				
Words understood	14.8/35.9	14	3.22 (1.49–6.94)	0.002
Words produced	16.0/33.3	14	2.61 (1.22–5.58)	0.009
Words understood	18.8/37.3	18	2.77 (1.23–6.28)	0.01
Words produced	19.1/37.3	18	2.01 (0.89–4.54)	0.07
**Toddler CDI** 2				
Words produced	17.1/35.0	18	2.60 (1.15–5.89)	0.02
**BSID-III** 3				
Receptive language	20.5/36.5	18	2.23 (1.08–4.60)	0.03
Expressive language	24.1/37.5	18	1.89 (0.94–3.83)	0.08
Cognitive	23.1/20.0	18	1.20 (0.55–2.60)	0.70
Fine Motor	25.6/30.1	18	1.25 (0.61–2.55)	0.33
Gross Motor	26.6/29.7	18	1.17 (0.58–2.37)	0.40

^1^ High scores were defined as those scores achieved by the highest performing infants whereby n had the lowest possible deviation from 25% of all infants, by sex. Odds ratios and 5^th^-95^th^ confidence interval (CI) determined using contingency tables with *P* values determined using Fisher exact analysis.

^2^ CDI, MacArthur Communicative Development Inventory.

^3^ BSID, Bayley Scales of Infant Development III.

## Discussion

The present study uses a risk-reduction model to assess whether some infants fail to meet their developmental potential due to insufficient DHA in gestation to meet the needs of the developing CNS. Our study does not address the reasons for inadequate DHA, does not identify dietary requirements, and does not aim to show that supplemental DHA enhances cognitive abilities. However, using a model designed to identify DHA insufficiency in the infant before birth, we provide novel data to show that DHA low enough to limit infant development to 18 mo of age does occur. In the discussion, we highlight the complexity of identifying n-3 fatty acid deficiency before birth, and raise several points for studies designed with this intent.

In our study, women were enrolled at 16 wk gestation, and randomized to DHA or a placebo until delivery of the infant for the purpose of assessing if fetal CNS development is constrained among infants of women following their usual diet (Figure 1). Pragmatically, since DHA consumed by the mother is transported across the placenta [51], [52] and provides DHA to the fetal CNS [19]–[22], it is expected that maternal DHA supplementation should reduce risk of insufficient fetal DHA. This concept is consistent with observation studies to show lower risk of poor child CNS development in infants and children of women consuming diets rich in fish or marine mammals [23]–[26]. However, the corollary that women with low intakes of DHA necessarily equate with fetal DHA inadequacy cannot be assumed from current knowledge. Data to show poor neurodevelopment in children of healthy vegetarians, despite their low diet and blood lipid DHA [53] has not been published. Variable maternal-to-fetal fatty acid transfer, and fetal n-3 fatty acid metabolism and acylation into neural tissue may also limit extrapolation from measures of maternal DHA to DHA accretion in the fetal CNS. Recognition that assessment of fetal DHA insufficiency differs from assessment in breast-fed and formula-fed infants, children or adults in whom blood levels of DHA can be directly measured is important.

We randomized pregnant women to take four to ten fold more DHA than the estimated fetal DHA needs [40], [41], then assessed risk that healthy term gestation infants born to mothers given a placebo would fail to be among the quartile of infants achieving the highest CNS development (Figure 1). Using this approach, we show increased risk of lower language development at 14 and 18 mo of age on the CDI infant and toddler scales (*P*<0.02) and on the BSID-III (*P*<0.05) at 18 mo of age among infants in the placebo group. Constraint of language development was robust, affecting both boys and girls, productive and receptive language, and evident in well-controlled testing using the BSID-III with testers blinded to the infant group. Visual acuity also showed evidence of delayed maturation in the placebo group when assessed at 2 mo (OR 2.69, CL 1.10–6.54, *P* = 0.03), but not at 12 mo of age. Previous prospective observation studies [23], [48] and clinical trials with preterm infants given DHA [54] have also found language development appears to be sensitive to the early DHA supply. Possibly, these results reflect a role of DHA in the anatomical and molecular factors involved in early language learning. It is of interest that whereas auditory stimuli elicit large event related potentials over temporal brain regions with absent or small responses over occipital regions in the adult brain, 6 mo old infants show equally large response amplitudes over the visual and auditory cortex on auditory stimulation [55]. Maturation and increasing specificity of auditory sensory systems, fundamental to developmental sensitivities of grammatical and semantic/lexical (object-word association) language learning, involve a gradual decrease in responses of occipital regions to auditory stimuli from 6 to 36 mo of age. Although apparently not studied in humans, experimental work has reported evidence of altered auditory evoked potentials in offspring of animals given DHA during pregnancy [56]. We found no evidence of fetal DHA insufficiency sufficient to constrain cognitive, gross motor or fine motor development when assessed using the BSID-III at 18 mo of age. The categorical pass/fail results for problem solving and non-native language tests found no group differences, however, tests which do not enable ranking of infant skill development may lack the sensitivity to detect slower rates of CNS development in term infants.

Several studies have addressed the efficacy of maternal prenatal DHA supplementation as a means to increase infant and child neurodevelopment, with findings of no benefit on novelty preference at 6 or 9 mo [27], a small increase (*P* = 0.049) in mean scores on the mental composite scale of the Kauffman Assessment Battery for Children (KAB-C) at 4 y but not at 7 y [28], [29], significant positive effects on group mean problem solving, but not novelty preference at 9 mo [31], and no significant difference in group mean test scores on the BSID-III at 18 mo [36], [37], Griffiths Mental Developmental Scale or Picture Peabody Test at 2.5 y [32], the Hemple, Touwen, or KAB-C tests at 4, 5.5 or 6.5 y of age, respectively [34], [35]. In our study, we also found no significant difference in group mean test scores between infants in the placebo and DHA groups for any test, at any age. Our results thus concur with a conclusion that at a group level, supplemental DHA does not enhance cognitive development of infants born at term gestation [37]. Assuming no random group bias, our results for language development using the BSID III estimate constraint of development in about 10% of infants in the placebo group, a number too small to detect by group mean comparisons with our sample size enrolled without a priori knowledge of deficiency.

Studies in other species have shown that n-3 fatty acid deficiency results in increased tissue lipid 22:4n-6 and 22:5n-6 [5], [13], [14], [22]. Experimental evidence for a pregnancy-associated increase in hepatic Δ 6 desaturase gene expression has been reported [57]. Our results show increased 22:4n-6 + 22:5n-6, but not DHA, in RBC PE with advancing gestation in women in the placebo group (Figure 3). We interpret this as biochemical evidence of insufficient n-3 fatty acids to support the gestational increase in fatty acid desaturation, which thus proceeded with n-6 fatty acids. Whether this was due to insufficient n-3 fatty acids, excess n-6 fatty acids or some other factor is unclear. We found no significant relationship between the maternal RBC DHA at 16 or 36 wk gestation and infant CNS development. Regardless of the positive correlation between the maternal DHA intake and the RBC PE and PC DHA (*P*<0.001), the RBC DHA showed considerable variability among women with similar DHA intakes (Figure 3). The RBC DHA levels also overlapped between women in the placebo and DHA group at 36 wk gestation after about 20 wk supplementation (Figure 3). Other studies also show high variability in blood lipid DHA and in the response to DHA supplementation. For e.g., the mean ± 2SD range of plasma phospholipid DHA was 50–153.6% and 79–215.4% fatty acids in 35 wk gestation women assignment to placebo or 1183 mg DHA + 803 mg 20:5n-3/d from 17–19 wk gestation, respectively [52], and the RBC PE DHA was 2.92–10.2% and 3.46–14.1% fatty acids in term gestation women given a placebo or 500 mg DHA + 150 mg 20:5n-3/d from 20 wk gestation [58]. Although the reasons for the overlap in blood lipid DHA among women with widely different DHA intakes is not known, it is evident that DHA acylation into blood lipids is more complex than DHA intake. Similar complexity in extrapolating DHA accretion in the fetal CNS from measures of the exogenous, i.e. maternal DHA supply, would seem likely.

This study has several important limitations. High breast-feeding rates, with 63–72% infants still breast-fed at 6 mo may have masked greater constraint of CNS development in infants with shorter exposure to or no breastfeeding. Brain DHA accretion and neural synapse development continues throughout early childhood. Postnatal compensation or other nutrient deficits would dampen any effect of fetal DHA insufficiency on CNS development assessed after birth. Our definition of failure to be among the quartile of infants with the highest developmental test scores was arbitrary, and different definitions would yield different results. Although this study was randomized, it is possible that unmeasured variables in the home environment were present between the groups and contributed to the differences found. Regardless, this study was designed with a randomized intervention in a quasi-risk reduction model to provide evidence of fetal DHA insufficiency in our population. Using this approach, we have shown that language development is robustly constrained risk at different ages and with different tests in infants born to women consuming about 5% energy from 18:2n-6, 0.59% energy as 18:3n-3 and 85mg/d DHA. We emphasize that we do not hypothesize that DHA enhances child neurodevelopment, but our data support an hypothesis of DHA insufficiency among infants of women following typical western diets for which risk may be reduced by increasing the maternal DHA intake.

## Supporting Information

Figure S1 **Scatter plots to show the relationship between dietary DHA intake and the RBC PE DHA as g/100 g fatty acids in panels A and B, and RBC PC in panels C and D, at 16 wk gestation in panels A and C, and 36 wk gestation, panels B and D, for women not taking any supplemental DHA.** Correlation coefficients were calculated using Spearman's rho.
(TIF)

File S1 **Contains Tables S1–S4. Table S1.** Results are means ± SD (*n*) calculated using the WHO Anthroplus anthropometric calculator (version 1.0.4). There were no significant differences between the groups, by ANOVA, *P*≥0.05. **Table S2.** Results are medians (2.5-97.5^th^ percentile range) of fatty acid intakes. There were no significant differences in intakes between 16 and 36 wk gestation, or between the two randomized groups by Wicoxen signed-rank test. *^2^* % en, % total dietary energy. **Table S3.** *^1^* Results are means ± SD, g/100 g fatty acids. *^2^ P* value for differences between groups at the same stage of gestation, by ANOVA. *^3,4^* Value at 36 wk gestation different from 16 wk gestation within a group, *P*<0.01 or *P*<0.05, respectively, by ANOVA. **Table S4.** *^1^* Results are means ± SE, g/100 g fatty acids. *^2^ P* value for differences between groups at the same stage of gestation, ANOVA. *^3,4^* Value at 36 wk gestation different from 16 wk gestation within a group, *P<*0.01 or *P<*0.05, respectively, by ANOVA.
(DOCX)

Checklist S1 **CONSORT Checklist.**
(DOC)

Protocol S1 **Study Protocol.**
(PDF)
