# Supplementary figures and images for: Omega-3 Fatty Acid Deficiency in Infants before Birth Identified Using a Randomized Trial of Maternal DHA Supplementation in Pregnancy
Source: PLoS One. 2014 Jan 10;9(1):e83764. doi: 10.1371/journal.pone.0083764 (PMC3888379; doi:10.1371/journal.pone.0083764)

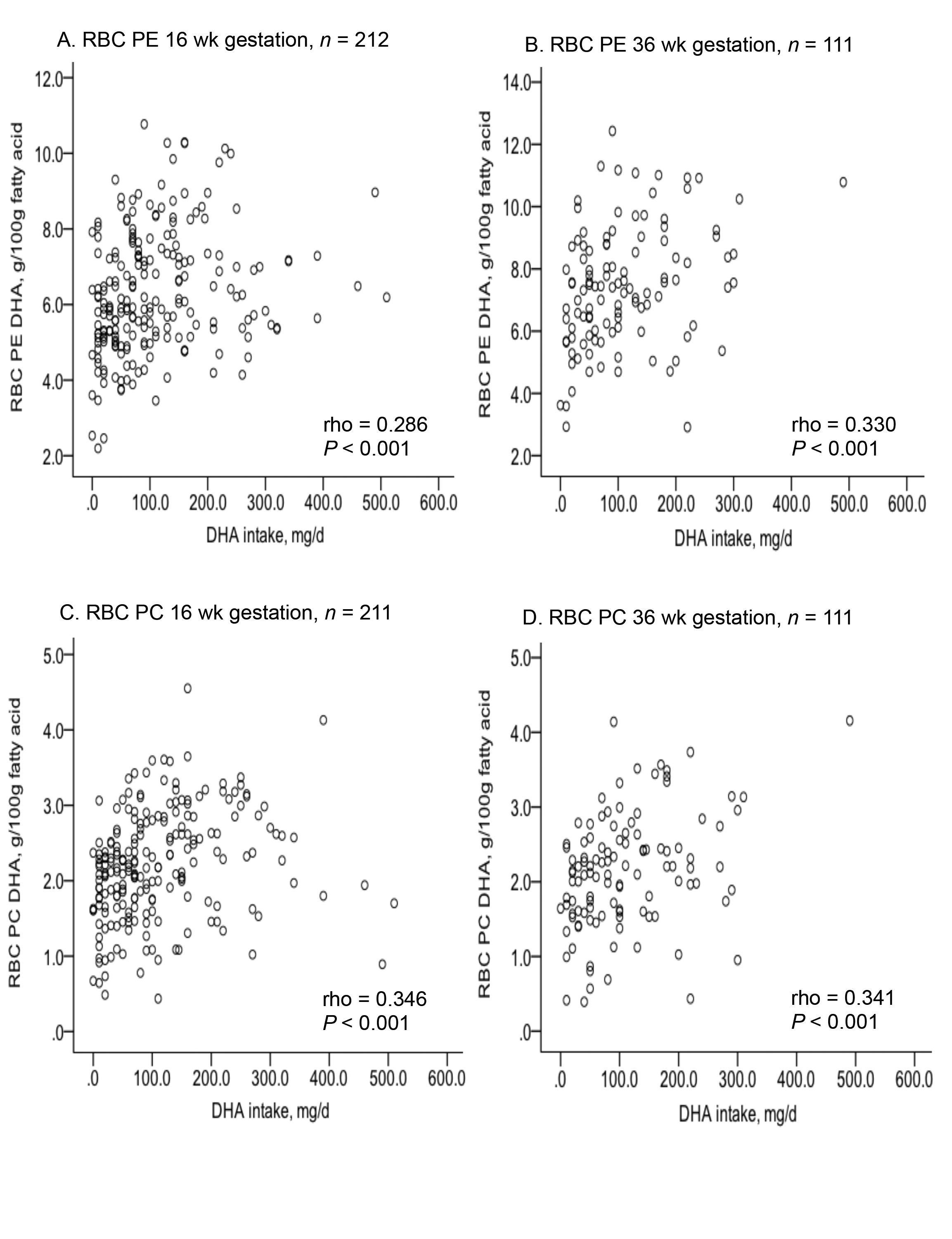

Supplement: Figure S1 — Scatter plots to show the relationship between dietary DHA intake and the RBC PE DHA as g/100 g fatty acids in panels A and B, and RBC PC in panels C and D, at 16 wk gestation in panels A and C, and 36 wk gestation, panels B and D, for women not taking any supplemental DHA. Correlation coefficients were calculated using Spearman's rho. (TIF) [file pone.0083764.s001.tif]
