# Supplementary material for: Omega-3 Fatty Acid Deficiency in Infants before Birth Identified Using a Randomized Trial of Maternal DHA Supplementation in Pregnancy
Source: PLoS One. 2014 Jan 10;9(1):e83764. doi: 10.1371/journal.pone.0083764 (PMC3888379; doi:10.1371/journal.pone.0083764)
Supplement: File S1 — Contains Tables S1–S4. Table S1. Results are means ± SD (n) calculated using the WHO Anthroplus anthropometric calculator (version 1.0.4). There were no significant differences between the groups, by ANOVA, P≥0.05. Table S2. Results are medians (2.5-97.5th percentile range) of fatty acid intakes. There were no significant differences in intakes between 16 and 36 wk gestation, or between the two randomized groups by Wicoxen signed-rank test. 2 % en, % total dietary energy. Table S3. 1 Results are means ± SD, g/100 g fatty acids. 2 P value for differences between groups at the same stage of gestation, by ANOVA. 3,4 Value at 36 wk gestation different from 16 wk gestation within a group, P<0.01 or P<0.05, respectively, by ANOVA. Table S4. 1 Results are means ± SE, g/100 g fatty acids. 2 P value for differences between groups at the same stage of gestation, ANOVA. 3,4 Value at 36 wk gestation different from 16 wk gestation within a group, P<0.01 or P<0.05, respectively, by ANOVA. (DOCX) [file pone.0083764.s002.docx]

**Table S1**

Anthropometric measures (z scores) of infants from 2 to 18 mo-of-age

|  |  | Maternal supplement group | |  |
| --- | --- | --- | --- | --- |
| Age | Measure | Placebo | DHA | *P* |
| 2 mo | Weight-for-length | -0.16 ± 1.08 (101) | -0.42 ± 1.20 (90) | 0.11 |
|  | Length-for-age | 0.29 ± 1.08 (102) | 0.17 ± 1.04 (92) | 0.42 |
|  | Weight-for-age | 0.06 ± 1.08 (101) | -0.19 ± 1.08 (90) | 0.11 |
| 6 mo | Weight-for-height | 0.04 ± 1.04 (101) | -0.11 ± 1.02 (95) | 0.32 |
|  | Height-for-age | 0.25 ± 1.06 (101) | 0.17 ± 1.04 (95) | 0.58 |
|  | Weight-for-age | 0.10 ± 1.01 (101) | -0.06 ± 1.11 (95) | 0.30 |
| 9 mo | Weight-for-length | -0.04 ± 0.99 (94) | 0.17 ± 1.05 (87) | 0.16 |
|  | Length-for-age | 0.22 ± 1.08 (95) | -0.06 ± 1.05 (88) | 0.08 |
|  | Weight-for-age | 0.03 ± 0.99 (94) | 0.04 ± 1.11 (87) | 0.92 |
| 12 mo | Weight-for-height | -0.04 ± 0.99 (93) | 0.14 ± 1.09 (81) | 0.24 |
|  | Height-for-age | 0.44 ± 1.11 (94) | 0.11 ± 1.06 (84) | 0.05 |
|  | Weight-for-age | 0.15 ± 1.02 (94) | 0.12 ± 1.05 (81) | 0.86 |
| 18 mo | Weight-for-length | 0.14 ± 1.05 (70) | 0.14 ± 1.05 (74) | 0.90 |
|  | Height-for-age | 0.41 ± 1.14 (82) | 0.16 ± 1.11 (76) | 0.17 |
|  | Weight-for-age | 0.27 ± 0.99 (70) | 0.21 ± 1.04 (74) | 0.71 |

*^1^* Results are means ± SD (*n*) calculated using the WHO Anthroplus anthropometric calculator (version 1.0.4). There were no significant differences between the groups, by ANOVA, *P* ≥0.05.

**Table S2.**

Dietary n-6 and n-3 fatty acids intakes among Canadian pregnant women at 16 and 36 wk gestation randomized to placebo or DHA supplement*^1^*

| **Fatty acid** | **Placebo** | | **DHA** | |
| --- | --- | --- | --- | --- |
|  | 16 wk  (*n* = 111) | 36 wk  (*n* = 111) | 16 wk  (*n* = 103) | 36 wk  (*n* = 103) |
| 18:2n-6, % en | 4.98 (2.32-10.3) | 5.26 (2.82-9.80) | 4.85 (2.96-8.71) | 5.31 (2.87-8.74) |
| 18:3n-3, % en | 0.59 (0.29-1.94) | 0.61 (0.31-1.69) | 0.59 (0.35-1.55) | 0.57 (0.35-1.61) |
| 20:4n-6, mg/d | 90.0 (20.0-204) | 90.0 (26.0-270) | 80.0 (26.0-208) | 90.0 (20.0-228) |
| 20:5n-3, mg/d | 40.0 (0.00-224) | 30.0 (0.00-160) | 50.0 (0.00-314) | 40.0 (0.00-162) |
| 22:6n-3, mg/d | 80.0 (10.0-334) | 90.0 (10.0-302) | 90.0 (6.0-472) | 100 (10.0-346) |
| 18:2n-6/n-3 | 7.64 (3.20-14.8) | 7.43 (3.58-14.4) | 6.84 (3.50-13.2) | 7.63 (3.97-13.4) |

*^1^*Results are medians (2.5-97.5^th^ percentile range) of fatty acid intakes. There were no significant differences in intakes between 16 and 36 wk gestation, or between the two randomized groups by Wicoxen signed-rank test.

*^2^* % en, % total dietary energy.

**Table S3**.

Major fatty acids in red blood cell phosphatidylethanolamines (g/100g fatty acids) of Canadian women at randomized to a placebo or 400mg/d DHA from 16 wk gestation*^1^*

| Fatty acid | Gestation wk 16 | | | Gestation wk 36 wk | | |
| --- | --- | --- | --- | --- | --- | --- |
|  | Placebo  *n* =111 | DHA  *n* =102 | *P*^2^ | Placebo  *n* =111 | DHA  *n* =104 | *P*^2^ |
| 16:0 | 14.8±1.79 | 14.8±2.20 | 0.88 | 15.7±2.23^3^ | 16.1±2.58^3^ | 0.18 |
| 18:0 | 7.15±1.43 | 7.11±1.33 | 0.84 | 6.67±1.15^3^ | 6.74±1.16^4^ | 0.64 |
| 18:1 | 15.3±1.38 | 15.3±1.20 | 0.95 | 16.7±1.73^3^ | 16.5±1.34^3^ | 0.35 |
| 18:2n-6 | 4.55±0.80 | 4.59±0.78 | 0.55 | 4.71±1.00 | 4.47±0.77^4^ | 0.05 |
| 20:3n-6 | 1.13±0.34 | 1.15±0.33 | 0.58 | 0.96±0.58 | 0.95±0.53^3^ | 0.93 |
| 20:4n-6 | 17.2±1.67 | 17.6±2.00 | 0.20 | 16.4±1.73^3^ | 15.7±2.02^3^ | <0.01 |
| 22:4n-6 | 5.40±1.10 | 5.41±1.32 | 0.92 | 5.46±1.24 | 4.46±1.01^3^ | <0.01 |
| 22:5n-6 | 0.63±0.20 | 0.65±0.27 | 0.76 | 0.82±0.24^3^ | 0.55±0.17^3^ | <0.01 |
| 18:3n-3 | 0.25±0.10 | 0.23±0.07 | 0.12 | 0.26±0.07^3^ | 0.24±0.06^4^ | 0.01 |
| 20:5n-3 | 0.80±0.37 | 0.76±0.27 | 0.86 | 0.69±0.30^3^ | 0.74±0.26 | 0.06 |
| 22:5n-3 | 3.12±0.61 | 3.13±0.55 | 0.90 | 3.16±0.68 | 2.63±054^3^ | <0.01 |
| 22:6n-3 | 6.24±1.60 | 6.36±1.63 | 0.60 | 7.44±1.94^3^ | 9.98±2.01^3^ | <0.01 |

*^1^* Results are means ± SD, g/100g fatty acids.

*^2^ P* value for differences between groups at the same stage of gestation, by ANOVA.

*^3,4^* Value at 36 wk gestation different from 16 wk gestation within a group, *P* <0.01 or *P* <0.05, respectively, by ANOVA.

**Table S4**.

Major fatty acids in red blood cell phosphatidylcholine at 16 and 36 wk gestation of women randomized to a placebo or 400mg/d DHA from 16 wk gestation*^1^*

| Fatty acid | Gestation wk 16 | | | | Gestation wk 36 wk | | |
| --- | --- | --- | --- | --- | --- | --- | --- |
|  | Placebo  *n* =111 | DHA  *n* =102 | | *P*^2^ | Placebo  *n* = 111 | DHA  *n* =104 | *P*^2^ |
| 16:0 | 36.2±2.22 | | 36.2±1.60 | 0.77 | 37.6±1.63^3^ | 37.7±1.40^3^ | 0.61 |
| 18:0 | 10.1±0.95 | | 9.96±0.89 | 0.32 | 8.69±0.75^3^ | 8.73±0.71^3^ | 0.76 |
| 18:1 | 19.1±1.95 | | 18.6±1.16 | 0.06 | 18.7±1.37 | 18.6±1.54 | 0.68 |
| 18:2n-6 | 19.9±2.15 | | 20.5±1.96 | 0.41 | 20.7±2.43^3^ | 20.6±2.16 | 0.76 |
| 20:3n-6 | 2.63±0.55 | | 2.67±0.66 | 0.64 | 2.67±0.47 | 2.56±0.50^4^ | 0.07 |
| 20:4n-6 | 5.81±1.27 | | 5.80±1.26 | 0.99 | 5.36±1.13^3^ | 5.04±1.09^3^ | 0.03 |
| 22:4n-6 | 0.34±0.32 | | 0.32±0.12 | 0.98 | 0.28±0.10^4^ | 0.23±0.08^3^ | <0.01 |
| 22:5n-6 | 0.17±0.09 | | 0.17±0.09 | 0.85 | 0.21±0.11^3^ | 0.14±0.08^3^ | 0.85 |
| 18:3n-3 | 0.34±0.09 | | 0.34±0.18 | 0.06 | 0.38±0.11^3^ | 0.34±0.10^3^ | 0.01 |
| 20:5n-3 | 0.40±0.26 | | 0.38±0.16 | 0.98 | 0.32±0.16^3^ | 0.37±0.20 | 0.06 |
| 22:5n-3 | 0.43±0.15 | | 0.41±0.11 | 0.66 | 0.36±0.12^3^ | 0.32±0.24^3^ | <0.01 |
| 22:6n-3 | 2.19±0.76 | | 2.21±0.68 | 0.96 | 2.13±0.78 | 2.82±0.87^3^ | <0.01 |

*^1^* Results are means ± SE, g/100 g fatty acids.

*^2^ P* value for differences between groups at the same stage of gestation, ANOVA.

*^3,4^* Value at 36 wk gestation different from 16 wk gestation within a group, *P<*0.01 or *P<*0.05, respectively, by ANOVA.
