# Supplementary material for: Omega-3 Fatty Acid Deficiency in Infants before Birth Identified Using a Randomized Trial of Maternal DHA Supplementation in Pregnancy
Source: PLoS One. 2014 Jan 10;9(1):e83764. doi: 10.1371/journal.pone.0083764 (PMC3888379; doi:10.1371/journal.pone.0083764)
Supplement: Protocol S1 — Study Protocol. (PDF) [file pone.0083764.s004.pdf]

Protocol

N-3 FATTY ACID REQUIREMENTS FOR HUMAN DEVELOPMENT

DR. SHEILA M. INNIS
DEPARTMENT OF PEDIATRICS
UNIVERSITY OF BRITISH COLUMBIA

5/22/03 10:31 AM

Study Objectives

Compare the effects on CNS maturity, language and cognitive development, growth, and essential fatty acid status as well as serum parameters in term infants following maternal supplementation during gestation. The study supplements are:

1) Single cell triglyceride capsules to provide about 400mg 22:6n-3 containing an orange flavor.
2) Soybean/corn oil as placebo capsules containing an orange flavor.

I. CNS maturity:

Compare CNS maturity and infant blood lipid 22:6n-3 with and without an increase in the maternal intake of 22:6n-3 of 400mg/day. Compare EEG maturity, ABR amplitude and latency 48-72 hr, 3 months of age, as well as looking acuity at 2, 3 and 12 months of age.

II. Language development:

Compare speech perception at 9 months and vocabulary comprehension and production at 14 and 18 months.

III. Sleep/Wake patterns:

Compare sleep/wake cycles over a 48-hour period from weeks 4 to 8, and over a 24-hour period at weeks 10, 12, 16 and 18.

IV. Cognitive and behavioral development:

Compare infant mental and physical development at 9 months and 18 months of age and behavioral development at 6, 12 and 18 months of age.

V. Growth:

Compare growth by measuring participants weight, length and head circumference at birth, then at 1,2,3,6,9,and 18 months using standardized procedures.

VI. Nutrient intake of mothers and infants:

Compare energy, fat and nutrient intake of mothers by using a food frequency questionnaire along with a weighed 3-day record at 16 and 36 week gestation. Compare nutrient intake of infants using a feeding diary every month and an infant food frequency questionnaire at 9 & 18 months of age.

VII. Lipid and fatty acid analysis:

Compare maternal blood collected at 16 and 36 weeks gestation and infant blood at 2 months for fatty acid analysis. Compare fetal cord blood and umbilical cord tissue following delivery of the infant. Compare human milk from breast-feeding mothers at 1, 2 and 3 months post-partum.

Study design

I. Description of study:

This will be a randomized, blinded, prospective study with 2 groups; placebo group and supplemented group with 22:6n-3. Women will be enrolled and randomized by 16 weeks gestation. An initial dietary assessment will be done and blood will be collected at 16 weeks. The dietary supplementation will then commence and continue until delivery. The dietary assessment and maternal blood sample will be repeated at 36 weeks gestation. Hospital registration charts will be marked to indicate the women who are participants in this research, and will contain a copy of the informed consent. Following delivery, fetal cord blood and umbilical cord tissue will be collected. Length of gestation, newborn infant weight, length, head circumference and gender will be recorded. The mother-infant pairs will be followed for 18 months following delivery.

To account for drop-outs, approximately 200 mothers per group will be randomized in order to have 120 participants per group.

II. Primary/Secondary Endpoints

Primary: Infant CNS maturity

Secondary: language development, sleep-wake cycle and crying behavior, cognitive and behavioral development, growth (weight, length, head circumference), maternal lipid and fatty acid analysis, gestation length and incidence of prematurity.

Selection of participants

I. Study population:

Acceptable participants will be identified from the registrations for low-risk delivery at the BC Women's Hospital and through advertising. Eligible participants will be 20-40 years of age, 12-16 weeks gestation, expected to deliver a single full-term (37-42 weeks) infant.

II. Participant Exclusion Criteria

All mothers:

- Diabetes
- Cardiac disease
- Renal disease
- Tuberculosis
- HIV/AIDS
- Hepatitis
- Previous pregnancy complications
- Substance abuse

III. Randomization

Participants will be randomized to the supplements using a random number system. The supplements will be given as 2x500mg capsules of single cell triglycerides to provide about 400 mg 22:6n-3, or soybean/corn oil as a placebo. The supplements and placebo capsules will be provided in prepackaged sealed containers for dispensing and will both contain an orange flavor to assist in further blinding. The capsules will be dispensed at 16, 26 and 36 weeks. The number of capsules remaining in each vial will be recorded, and used with the number dispensed, to calculate the number taken by each subject.

Investigational Plan

Study Procedures and Observations

Study visits will occur when the participants are at 16 and 36 weeks gestation, and at 1, 2, 3, 6, 9, 12, 14 & 18 months postpartum.

Study visit 1 (16 weeks gestation)

- Informed consent – At or prior to this visit, the participant who meets all of the applicable inclusion criteria and none of the exclusion criteria will be contacted and the study will be explained to them. Their questions will be answered to their satisfaction. After giving time for consideration, the participant will be asked to grant permission for their infant and themselves to participate in the study. If permission is granted, an informed consent form will be signed by the participant and a copy of the signed consent written informed consent form will be provided to them.
- Verify inclusion and exclusion criteria.
- Randomization.
- Dispense supplements.
- Dietary assessment (Food frequency of prior 4 weeks + weighed 3 day food record)
- Maternal blood sample.

Study days 16 – 36 weeks gestation

- Periodic phone calls to participant.
- Dispense supplements.

Study visit 2 (36 weeks gestation)

- Dietary assessment (Food frequency of prior 4 weeks + weighed 3 day food record).
- Maternal blood sample.
- Dispense supplements.

Study days 36 weeks gestation – Day of delivery

- Periodic phone calls to participant.

Day of delivery (37 – 42 weeks gestation)

- Fetal cord blood collection.
- Anthropometric measurements of newborn (body weight, body length and head circumference).
- CNS maturation measures at 48-72 hr (EEG and ABR).

Study visit 3 (1 month postpartum)

- Breast-milk collection (breast-feeding mothers).
- Anthropometric measurements of infant (body weight, body length and head circumference).
- Infant feeding diary.

Study visit 4 (2 months postpartum ± 2 days)

- Breast-milk collection (breast-feeding mothers).
- Anthropometric measurements of infant (body weight, body length and head circumference).
- Looking acuity assessment.
- Infant feeding diary.

Study visit 5 (3 months postpartum ± 2 days)

- Breast-milk collection (breast-feeding mothers).
- Anthropometric measurements of infant (body weight, body length and head circumference).
- CNS maturation measures (EEG and ABR).
- Looking acuity assessment.
- Infant feeding diary.

Study months 4 & 5 postpartum

- Infant feeding diary.
- Periodic phone calls to participant.

Study visit 6 (6 months postpartum ± 2 days)

- Anthropometric measurements of infant (body weight, body length and head circumference).
- Infant characteristic questionnaire - ICQ (Bates)
- Infant feeding diary.

Study months 7 & 8 postpartum

- Infant feeding diary.
- Periodic phone calls to participant.

Study visit 7 (9 months postpartum ± 3 days)

- Anthropometric measurements of infant (body weight, body length and head circumference).
- Infant food frequency questionnaire (dietary intake).
- Language development (speech perception).
- Mental development (2 step problem solving)

Study months 10 & 11 postpartum

- Infant feeding diary.
- Periodic phone calls to participant.

Study visit 8 (12 months postpartum ± 5 days)

- Looking acuity assessment.
- Infant behavior questionnaire – IBQ (Rothbart)
- Infant feeding diary.

Study month 13 postpartum

- Infant feeding diary.
- Periodic phone calls to participant.

Study visit 9 (14 months postpartum)

- Language development (MacArthur CDI, infant version).
- Infant feeding diary.

Study months 15 to 17 postpartum

- Infant feeding diary.
- Periodic phone calls to participant.

Study visit 10 (18 months postpartum ± 5 days)

- Anthropometric measurements of infant (body weight, body length and head circumference).
- Infant food frequency questionnaire (dietary intake).
- Language development (MacArthur CDI, infant & toddler version).
- Early childhood behavior questionnaire – ECBQ (Bates)
- Bayley's Scales of Childhood Development (II).

Methods to assess study endpoints

1) Central Nervous System maturity

Electrophysiological measures, such as EEG and auditory evoked brain stem responses, will be used to assess the effects of 22:6n-3 on CNS maturation. All recordings will use routine scalp electrodes, and record per standard protocol.

The EEG will be recorded using 4 gold cup electrodes placed at sites Oz, O1, O2 and Pz per the International 10 – 20 System. For EEG backround, we record both light and dark conditions simulating open and eyes closed. ABR will be measured as described by Norcia & Tyler, duplicated for visual stimuli, and gated to the EEG recordings at the stated scalp sites (Oz, O1, O2, Pz). A Power Macintosh computer will drive a 20" high brightness monitor using custom software. A Neuroscan EEPEP40 system will record the electrophysiological signals and store the data. A swept parameter technique with black and white horizontal square wave gratings will be used and displayed on the monitor. Infants will be seated on their parent's lap in a darkened room, 100 cm from the display. VEP acuity estimates will be accepted as valid only if there are at least 3 points and the linear regression line is significant ($P<0.05$). Dr A Norcia will provide his software and training in this part of the study. Data analysis will include measures of EEG maturity, VEP amplitude and latency, and estimates of visual acuity.

Looking acuity will be assessed using the Teller Acuity Card Procedure at 2, 3 and 12 months of age in a dedicated room by a trained tester. Looking acuity will be determined as the finest grating to which the infant shows a reliable and consistent fixation response, based on the infant's looking behavior using a test distance of 36 cm at 2 and 3 months of age, and 56 cm at 12 months of age.

2) Language development

The tests of language development will use speech perception at 9 months postpartum and the MacArthur Communicative Developmental Inventory (CDI) at 14 & 18 months postpartum.

Speech perception

Speech perception is based on the ability of the infant to discriminate fine phonetic differences that distinguish syllables in the native and an unfamiliar language. This procedure involves conditioning the infant to provide an operant head-turn in response to a change in phonetic category within a series of speech stimuli. The infants will be tested in a sound attenuated chamber first on their ability to discriminate English voiced bilabial versus alveolar phones, followed by a non-English (Hindi) retroflex versus dental phonetic contrast. The Hindi language distinguishes four places of articulation (bilabial, dental, retroflex and velar), while the English language distinguishes 3 (bilabial, alveolar and velar). The test will be administered with the guidance of Dr J Werker. Data will be recorded for all infants of 9 months of age, but not analyzed for infants exposed to Hindi in their home environment.

McArthur CDI for infants and toddlers

The MacArthur CDI is a standardized parent report instrument designed to gather information on the infant's vocabulary comprehension and production. The infant version (words and gestures) has been developed and validated for infants 8 to 16 months of age and includes 396 words. The toddler version (words and sentences) is for infants 16 to 30 months of age. We will administer the infant CDI at 14 months postpartum and both the infant and toddler CDI at 18 months postpartum. The CDI results will be scored for the raw number of words, and the percentile scores for each age and gender (girls tend to be slightly advanced in gender).

3) Cognitive development

The Bayley Scales of Infant Development II, a standardized test of infant development, will be given at 18 months postpartum, with the mental development index (MDI) and psychomotor development index (PDI) standardized for age, based on reference norms. The MDI score is derived from the total number of items correctly performed, regardless of skill or weakness in any particular area. The Bayley Scales II, however, includes clusters of items (Facets) for the mental scale that provides information on language, cognitive and visual-motor abilities. Scores for each facet will be quantified as the ratio of correct items to the total number of items in the Facet as described by Grunnau. Scores on the Facets at 18 months of age are related to cognitive, verbal and visual-motor function at 4 – 5 years of age assessed with the Wechsler Preschool and Primary Scale of Intelligence. Dr. R Grunau, who has extensive experience with the Bayley Scales, will collaborate. The test will be given by a trained tester, with equipment and procedures. We will test at 18 months because the range of tasks that can be completed is greater than at younger ages, thus allowing a greater sensitivity to discriminate differences among infants.

4) Blood, tissue and milk samples

Blood will be drawn via venipuncture for a fatty acid profile from all mothers at 16 and 36 weeks gestation. A total of 3.0 ml of blood is needed for each blood draw. Fetal cord blood will be collected following delivery of the infant.

Breast-milk samples will be collected from all breast-feeding mothers at 1 and 2 months postpartum. The mothers will be instructed not to collect milk during the first 2 – 5 minutes of a feed (fore-milk), or drip milk, then interrupt the infant and gently express about 10 ml of human milk into the vial provided. The mother should label the vial with the date the sample was obtained. Samples will be frozen following collection, transported on ice and stored at -80 C on receipt at the lab.

5) Body weight

Birth weight will be obtained from the participant's birth records or directly from the parent/care giver, if the record is not available to the Investigator.

During the study period, infants will be weighed one time at each visit without clothing or diaper, on a standard pediatric balance, to the nearest gram. All balances will be checked to register the same weights throughout the range of weights expected.

6) Body length

Body length at birth will be obtained from the infant's birth records or directly from the parent/care giver, if the record is not available to the Investigator.

Body length for the study period will be measured 1 time at each visit to the nearest millimeter. The infant should be held in a recumbent position with the help of two examiners and a suitable measuring device. One person holds the infant's head into contact with the fixed headboard and a second person holds the infant's feet, toes pointing directly upward and while applying gentle traction, bring the movable footboard to rest firmly against the infant's heels.

7) Head circumference

Head circumference at birth will be obtained from the infant's birth records or directly from the parent/care giver, if the record is not available to the Investigator.

Head circumference for the study period will be measured 1 time at each visit to the nearest millimeter employing a flexible, non-stretchable cloth or vinyl tape. The tape is to be applied firmly around the head above the supraorbital ridges, covering the most prominent part of the frontal bulge anteriorly, and over the part of the occiput that gives maximum circumference.

8) Periodic phone calls

Study personnel will contact the participants periodically between study visits to collect information on diet, tolerance, and adverse events, determine if additional supplements are needed, remind participants of upcoming study visits, and answer questions concerning the study.

9) Maternal dietary intake/Infant dietary intake

At study visits 1 & 2, participants will be asked to complete a food frequency questionnaire of the preceding 4 weeks, together with a weighed 3-day food record. The food frequency questionnaire will be completed using an interview format with a trained dietician, using food models, scales, cups and spoons. Information on the frequency with which the food is eaten, portion size, brand name or place of purchase, methods of preparation, types of margarine, fats and oils, and types of fish and seafood will be collected.

Infant dietary intakes will be recorded using a feeding diary completed each month, and designed to capture when human milk is substituted, juices and solids are introduced and what they are. At study visits 7 and 10, dietary intake will be collected using an infant food frequency questionnaire.

10) Final evaluation

This form will document if the participants completed the study (visit 10) and if not, the reason for withdrawing from the study including the date of the last study visit.

Investigational product

The study supplements for the participants enrolled in this study will be provided in a capsule form.

These study supplements will be supplied by Martek Bioscience Corp:

- Single cell triglyceride capsules to provide about 400mg 22:6n-3.
- Soybean/corn oil as placebo capsules

The 22:6n-3 and placebo capsules will be provided in prepackaged sealed containers for dispensing. The participants will be randomized to the supplements using a random code number system. The study supplements will contain an orange flavor to assist in further blinding. The number of unused capsules will be recorded at 26 weeks gestation, 36 weeks gestation and after delivery in order to calculate the total number taken by each subject.

Duration of use
Study supplement usage is from 16 weeks gestation to day of delivery.

Statistics

Sample size
The calculation of sample size is based on the measures of CNS maturity of language development, VEP visual acuity and Bayley Scales, rather than the biochemical determinants, which will require a smaller sample size of <30 per group. Sample sizes were estimated for VEP acuity, language development at 9 and 14 months using the non-native consonant recognition and CDI, and Bayley Scales at 18 month. EEG was not included since there is no baseline information on which to estimate the difference or variability. Taking into account the multiple outcomes using the Bonferroni correction, the largest sample size required is 120/group with a corrected α of 0.05 and power of 80% to detect an effect size equal to half a standard deviation.

Statistical analysis
Differences in maternal plasma, RBC, fetal umbilical cord plasma and RBC and milk fatty acids between the 2 randomized groups will be determined using one way analysis of variance (ANOVA). One way ANOVA will be used to test for differences in measures of CNS maturity, looking acuity, and language development. Regression analyses will be used to determine the relation between intake (g/day & adjusted for energy intake) of selected fatty acids and the maternal and infant blood, umbilical tissue and milk 20:4n-6, 20:5n-3 and 22:6n-3. A stepwise multiple regression will be used to predict independent factors, including gender, maternal smoking and alcohol intake (as appropriate), highest level of education attained by the mother, family income, birth order, ethnic background, and maternal weight gain in pregnancy, maternal dietary

variables, and duration of breast-feeding, associated with the developmental outcomes. The measures of weight, length and head circumference will be used to plot growth curves for each infant, then an imputation procedure used to estimate weight, length, head circumference and rate of growth from birth to exactly 4, 6, 9 and 18 month for each infant.
